# Shape Sensing with Rayleigh Backscattering Fibre Optic Sensor

**DOI:** 10.3390/s20144040

**Published:** 2020-07-21

**Authors:** Cheng Xu, Zahra Sharif Khodaei

**Affiliations:** Department of Aeronautics, Imperial College London, London SW7 2AZ, UK; z.sharif-khodaei@imperial.ac.uk

**Keywords:** fibre optic sensors, Rayleigh backscattering sensors, shape sensing, structural health monitoring

## Abstract

In this paper, Rayleigh backscattering sensors (RBS) are used to realize shape sensing of beam-like structures. Compared to conventional shape sensing systems based on fibre Bragg grating (FBG) sensors, RBS are capable of continuous lateral sensing. Compared to other types of distributed fibre optic sensors (FOS), RBS have a higher spatial resolution. First, the RBS’s strain sensing accuracy is validated by an experiment comparing it with strain gauge response. After that, two shape sensing algorithms (the coordinate transformation method (CTM) and the strain-deflection equation method (SDEM)) based on the distributed FOS’ input strain data are derived. The algorithms are then optimized according to the distributed FOS’ features, to make it applicable to complex and/or combine loading situations while maintaining high reliability in case of sensing part malfunction. Numerical simulations are carried out to validate the algorithms’ accuracy and compare their accuracy. The simulation shows that compared to the FBG-based system, the RBS system has a better performance in configuring the shape when the structure is under complex loading. Finally, a validation experiment is conducted in which the RBS-based shape sensing system is used to configure the shape of a composite cantilever-beam-like specimen under concentrated loading. The result is then compared with the optical camera-measured shape. The experimental results show that both shape sensing algorithms predict the shape with high accuracy comparable with the optical camera result.

## 1. Introduction

The usage of composites in air-foil and wind turbine blades has dramatically reduced the weight of these structures and, meanwhile, increased their deflections largely. The extreme deflection of the A350XWB wing, whose span is 64.75 m, is about 5.2 m, while Boeing 787’s wing deflection can go up to 7.9 m for a span of 60.1 m. An in-flight break-up accident of NASA’s Helios wing in 2003 and the awareness that part of the failure was due to excessive wing deformations has led to an urgency to develop a robust real-time shape sensing methodology [1].

Although various sensing technologies exist for monitoring the geometrical shape of a wing structure, such as the strain sheet measuring method, the vision measurement method, the photo elastic method, the laser scan measuring method and the three-coordinate measuring method [2,3,4,5,6], their instalments require enormous space. They are only suitable for the ground testing of the wing shape and not applicable for the real-time monitoring of the aerodynamic shape of a morphing wing during flight [7].

Fibre optic sensors (FOS), due to their advantages such as immunity from electromagnetic interference, low weight and small size, which can be largely embedded in airplanes to form a sensor network, are generally viewed as the technology with the highest potential for the continuous real-time monitoring of aircraft structures by the scientific, industrial, and end-user communities [1]. The principle of shape sensing with the FOS system is to measure the strain along the structure and then configure the real-time shape via the strain-shape algorithm.

FOS can be classified into three main types: single point FOS, multi-point FOS and distributed FOS [1]. Most of the interferometric FOS are regarded as single point FOS. Although they have a higher strain resolution, their lack of a multiplexing capability limits their application in shape sensing. Grating-based FOS and backscattering-based FOS are regarded as multi-point and distributed FOS, respectively. Among them, fibre Bragg grating (FBG) has been researched the most in the last two decades. Various studies have been reported in the literature applying FBG in shape sensing. The NASA Armstrong Flight Research Centre (AFRC) has developed an instrumentation system and analysis techniques to realize the shape sensing of air-foil in lightweight vehicles [8,9,10]. Nicolas et al. [11] realized the shape sensing of the composite beam using FBGs based on the strain deflection equation method (SDEM), the algorithm originally developed by [12]. A structural shape measurement system using multi-core fibre sensing method with FBGs was developed by Childlers et al. [13]. The coordinate transformation method (CTM) was proposed by P. Ferreira et al. [14] for the shape sensing of a composite paddle using FBGs. In distributed FOS, the optical fibre itself becomes the sensor, by detecting changes in the characteristics of the light scattered along the fibre length caused by the local variation of physical quantities such as strain or temperature. There are three types of scattering: Rayleigh, Brillouin and Raman [1]. Rayleigh backscatter is an elastic physical phenomenon caused by random fluctuations in the reflection index profile along the length of the tested fibre. Brillouin scattering is an inelastic type of scattering caused by the interaction of sound waves traveling in opposite directions. Another inelastic type of scattering is Raman scattering, which is caused by the interaction of the light wave with molecular vibrations in the transmission medium. It is only sensitive to temperature and therefore cannot be used in strain measurement. For strain measurements, Rayleigh and Brillouin scattering are of current interest [1]. However, unlike RBS, Brillouin scattering sensors are mostly used for static measurements [1]. 

Compared to multi-point techniques that use FBG sensors for strain-based shape reconstruction [8,9,10,11,13,14], the main advantage offered by distributed sensing is the large availability of strain data, due to continuous lateral sensing over long fibre spans. This can therefore improve substantially the application of algorithms used for shape reconstruction based on strain measurements and makes it possible to take into account existing non-uniformities in the measured strain profiles, thus improving the accuracy of the shape reconstruction [1].

The main problem in distributed FOS’s application in shape sensing is their spatial resolution. There are different types of distributed FOS systems based on different principles or demodulation techniques. Most of them have a spatial resolution around the tens of centimetres. This leads to uncertainties in the accurate measurement location. The parameters of different distributed FOS systems’ spatial resolution are listed in Table 1. Currently, the only application of shape sensing with backscattering FOS is the work published by Nishio et al. [15], in which shape sensing with the Pre-pump pulse Brillouin Optical Time-Domain Analysis (PPP-BOTDA) technique has been carried out with a spatial resolution of 10 cm. The large spatial resolution reduced the measurement reliability of the Brillouin scattering based sensor (BBS), since the non-uniform strain distribution within BBS’ sensing parts leads to a distortion in the convex profile of each sensor in the Brillouin gain spectrum (BSG) and consequently influences the strain measurement reliability of the BBS sensor [15,16]. Although the continuous lateral sensing capability leads to a more integrated measured strain profile, the reliability of each sensing part is lower than the FBG sensors.

Most of the published work on shape sensing requires some assumptions for the strain distributions between two adjacent sensors, due to their low spectral resolution of strain profile and the discontinuity in sensing parts. Previous work related to continuous lateral sensing had a lower spatial resolution than FBG, which has led to a lower accuracy and robustness. Thus, the main objective of this work is to apply RBS in shape sensing and its targeted shape sensing algorithm based on coordinate transformation and strain-deflection relation, to improve current shape sensing system’s performance in predicting the deformed shape of beam-like structures under non-uniform loading.

In this paper, an RBS system with a spatial resolution of 2.5 mm is utilized in the shape sensing of composite beam-like structures. The paper first starts with the principle and the validation of the RBS system in strain sensing. It is then followed by different FOS strain-based shape sensing algorithms’ derivations and then optimizes the algorithms to increase their accuracy and robustness under complex and combine loading. The performance of the system is then validated through numerical simulation. Afterwards an experiment is conducted in which the RBS are bonded on one side of a composite cantilever-beam specimen to realize the real-time shape sensing of this structure. The measured result from the RBS-based shape sensing system is then compared with the results recorded by an optical camera.

## 2. Rayleigh Backscattering Sensing System

### 2.1. RBS System Architecture

**Figure 1:**
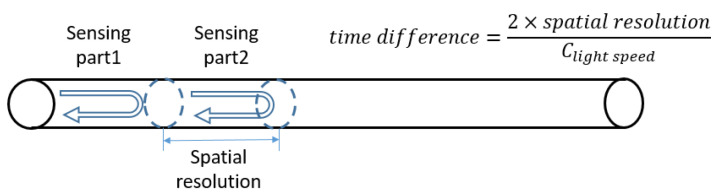
Principle of Optical Time Domain Reflectometry.

Previous RBS systems are based on the Optical Time Domain Reflectometry (OTDR) technique. In an OTDR system, two different sensing parts on an optical fibre are identified by the time difference between two beams of backscattering light (shown in Figure 1). Therefore, the photodetector’s sampling rate (around 1 GHz maximum) may not be high enough to distinguish between the signals from two adjacent sensing parts if the time interval between them is too small. This has led to the limitation of the minimum spatial resolution in OTDR systems. The large spatial resolution then causes uncertainty in accurate location measurement and decreases the number of sensing parts, i.e., sensors within one optical fibre.

**Figure 2:**
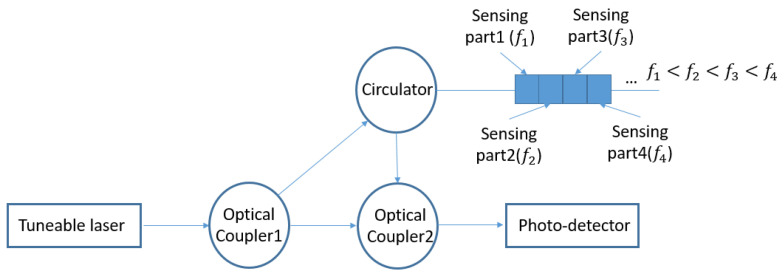
Schematic of an Optical Time Domain Reflectometry Rayleigh Backscattering Sensors System.

The Optical Frequency Domain Reflectometry (OFDR) technique has been developed to overcome this problem. The scheme of the OFDR-based RBS system is shown in Figure 2. The Tuneable Laser Source (TLS) irradiates a beam of light into the system, and the optical coupler1 separates the light into two parts: the probe and the reference light. The probe light goes through the FOS and receives Rayleigh backscattering light. The Rayleigh backscattering light interferes with the reference light at optical coupler2, the interferometric field which contains Rayleigh backscattering information is then recorded by the photodetector.

### 2.2. RBS Demodulation Principle

Rayleigh backscattering is caused by the random fluctuations in the index profile along the optic fibre. Each sensing part will be modulated by a unique frequency, the longer the distance between the sensing part and the laser is, the higher the frequency of its signal will be, see Figure 2. A strain or temperature change in a certain sensing part will lead to a frequency shift in the sensor part’s unique frequency. By calculating the cross-correlation between the testing and the reference signals in the frequency domain, the sensing part which is under loading can be identified. Through analysing the frequency shift within the light field received by the photodetector, the magnitude of the strain or temperature in that certain sensing part can then be demodulated. Due to the tuneable laser’s limitation in sweeping equal interval frequency and low optical intensity in backscattering light, the strain resolution for distributed FOS is lower than FBGs. For FBGs, each sensor has its own wavelength, even though the noise or transversal strain distribution within the optic fibre will lead to a distortion in the sensor’s spectrum, the FBG can still configure a relatively accurate average strain within its sensing part. By contrast, for distributed FOS, the sensing part is located by using cross-correlation between the test and reference signals, the distributed FOS can hardly configure any data from a distorted spectrum. Therefore, the robustness of the system is important for distributed FOS shape sensing.

### 2.3. RBS Spatial Resolution Selection in Shape Sensing

RBS’s spatial resolution is identified by the slide window length within its demodulation system. As mentioned in the previous section, the location of the sensing part is identified by calculating the cross-correlation between the testing and the reference signals in the frequency domain, the longer the length of the sliding-window, the more unique and identifiable are the characteristics contained in the subsets, and therefore the more reliable the results of the cross-correlation analysis. However, a longer sliding-window will lead to a lower spatial resolution and thus less data in the strain profile, which will cause an increase of errors in the shape sensing. In a scenario of single mode fibre (SMF) with refraction index 1.47 and light speed within the fibre 2.998 × 10^8^ m/s, the length between two adjacent point is around 9.578 μm. The data size based on Luna’s default spatial resolution (5 mm) [18] within each slide window will be over 500. This data size is definitely sufficient to offer a reliable cross-correlation analysis. However, research shows that a slide window with a data size around 300–400 can also perform strain sensing with sufficient reliability [25]. Other factors, including the nonlinear tuning of the light source, phase noise, polarization effects of the spectrum, noise in the measurement environment, as well as the error in bonding location will lead to a more severe error. Therefore, the spatial resolution used in this paper is 2.5 mm, which means around 270 data in each slide window.

### 2.4. RBS Strain Measurement Validation Experiment

The accuracy of strain sensing with RBS is validated first before its application in shape sensing, by carrying out an experiment in which RBS and strain gauges are bonded at positions with the same strain magnitude on a composite coupon. A linearly increasing load is then introduced onto the coupon. The strain measured by RBS and strain gauges under the same loading are recorded simultaneously.

**Figure 3:**
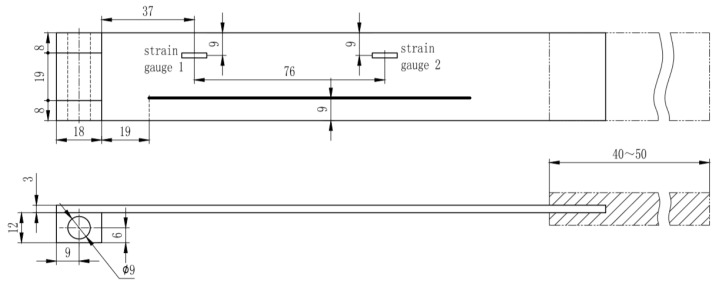
Specimen geometry and test set-up.

The carbon fibre reinforced polymer (CFRP) specimen used in this strain measurement validation experiment is manufactured from unidirectional prepreg (M21/194/34%/T800S (Hexcel)) with the layup of: [0/+45/−45/90/0/+45/−45/90] s. The size of the specimen is 250 × 35 × 3 mm^3^. Strain gauges are placed symmetrically about the centre line with respect to the RBS position. The demodulation system used in this experiment is a commercial system Luna ODiSI B with strain resolution 1 με. The spatial resolution for the RBS system used in this experiment is 10 mm, which is the same as the strain gauges’ length, so that no error is introduced due to the sensing length difference. One end of the specimen is fixed, while a concentrated loading is applied at the other end of the specimen. The location of the sensors as well as the loading and boundary condition are shown in Figure 3.

**Figure 4:**
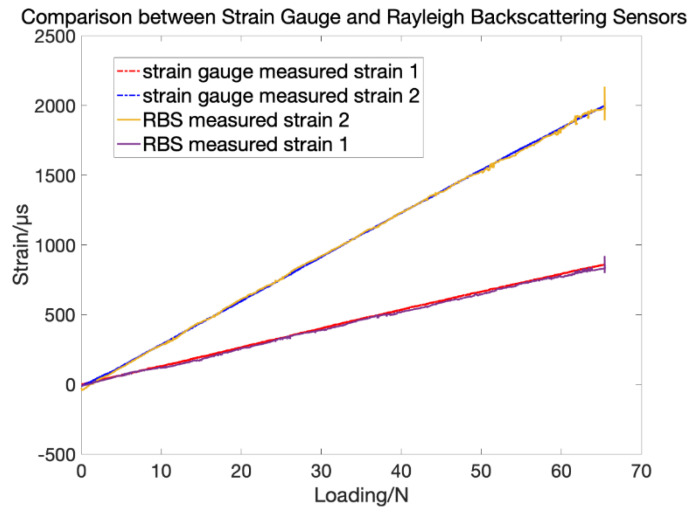
Strain measurement comparison between Rayleigh backscattering sensors and strain gauges.

The strain measurement results from both RBS and strain gauges are shown in Figure 4.

As can be seen, the strain measured by both RBS and strain gauges match perfectly. The difference between strain measured by strain gauge and RBS are within 5%, which validates that RBS can be used in strain measurement. In the next section, the strain-shape algorithm will be introduced so that the measured strain can then be used to reconfigure the deformed shape of the structure.

## 3. Strain-Shape Algorithm

The strain-deflection equation method (SDEM), alternatively called the “fibre optical sensing system method (FOSS)”, is the most popular method used in air-foil shape sensing. This algorithm relies on the classical beam theory to derive the displacement transfer functions in segments of the structure; details can be found in [10,12,26]. For completeness, the SDEM algorithm bases on discrete point sensing (FBG) and will be introduced in Section 3.1, and then shape sensing algorithms with distributed FOS will be presented in Section 3.2.

### 3.1. Shape Sensing Algorithms Based on Discrete Sensing

The derivation starts from Equation (1) which relates the vertical displacement W and the strain in optic fibre along the axial direction.

(1) $$\frac{d^{2}W}{dx^{2}}=\frac{\varepsilon \left(x\right)}{c\left(x\right)}$$

where c(x) is a parameter which equals to $\frac{2\Delta L}{T}$ when FOS are surface bonded. T represents the thickness of the beam which is constant. ΔL represents the length of each sensing part which can be adjusted to control the resolution of the algorithm. Once the FBG sensing network measures the strain on the sensing node, the strain distribution within each part is assumed as linearly distributed.

(2) $$\varepsilon \left(x\right)=\varepsilon_{i-1}-\left(\varepsilon_{i-1}-\varepsilon_{i}\right)\frac{x-x_{i-1}}{\Delta L}$$

Therefore, the deflection in each element can then be calculated as:

(3) $$y_{i}=\frac{{\left(\Delta L\right)}^{2}}{3T}\left[\left(3i-1\right)\varepsilon_{0}+6\overset{i-1}{\underset{j=1}{\sum }}\left(i-j\right)\varepsilon_{j}+\varepsilon_{i}\right],\left(i=1,2,3,\ldots ,n\right)$$

**Figure 5:**
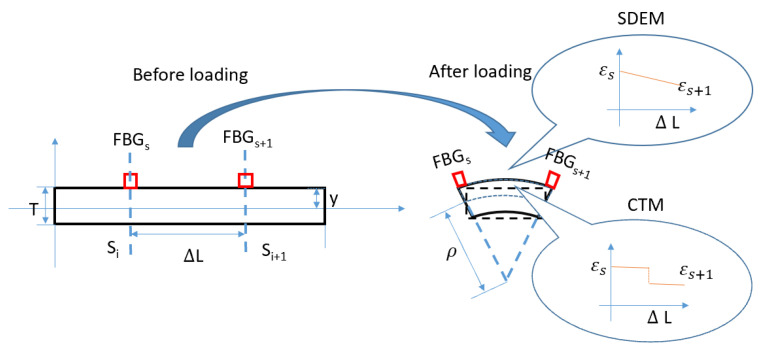
Element before and after loading.

Assuming a linear distribution for the strain in each sensing part translates that the structure is under a load distribution with concentrated force is applied at FBG’s location. This means that the external loading is concentrated forces located on the FBG’s measurement points. In the numerical simulation used in the patent [12] and in the experiments used in references [10] and [11] to validate this algorithm, the loadings are all in the form of concentrated force added on the location where FBGs are placed. In different shape sensing algorithms with discrete strain measurement, different strain distribution assumptions are made. In the coordinate transformation method mentioned in reference [14], the strain distribution is regarded as piece-wise uniform (shown in Figure 5). The CTM is initially used in sensing a large deformation. Thus, the rotation at each point is calculated as: $d\theta_{n}=2arctan\;\left(\frac{1}{2}\int_{s_{i}}^{s_{i+1}}k\left(s\right)ds\right)$ where $k\left(s\right)=\frac{1}{\rho }=\frac{\varepsilon_{n}}{T}$. *T* is the thickness of the structure. The calculated rotation in each sensing point is then divided equally and added to the rotation of the two adjacent elements. In a real situation, external loading is more likely to be a non-uniform distributed loading with concentrated force added on any place along the structure. In the next section, the SDEM and CTM in form of RBS-based shape sensing system is derived. Compared to the previous formula, no extra strain distribution assumption between two sensors are required.

### 3.2. Shape Sensing Algorithms Based on Continuous Lateral Sensing

**Figure 6:**
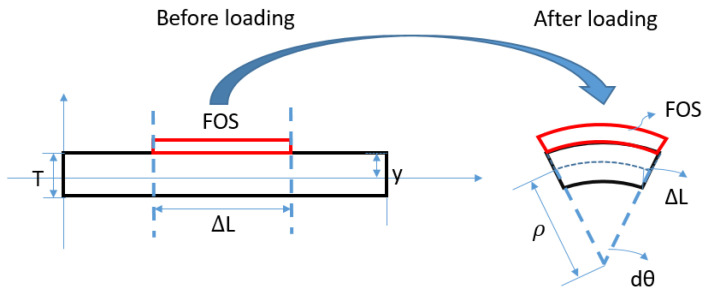
Element before and after loading for continuous fiber optic sensors.

**Figure 7:**
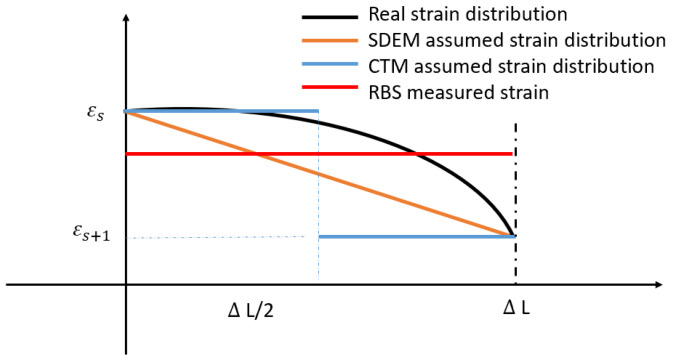
Strain distribution within different algorithms.

In this section, the algorithm for continuous strain sensing is introduced. Two methods (SDEM and CTM) for continuous lateral sensing is derived first and their performance for shape sensing is then compared and validated under various load distributions (e.g., non-uniform, partially loaded). In discrete sensing, FBG-measured data are regarded as the nodal strain in each element, while distributed FOS-measured data are the average strain within each part (shown in Figure 6). That means that within each element, strain on two ends of the part are measured in discrete sensing, while in continuous lateral sensing, only the average strain within the sensing part is achieved (shown in Figure 7). Thus, no extra strain distribution assumption is required. The strain through the whole structure is measured continuously.

#### 3.2.1. CTM for Distributed FOS

**Figure 8:**
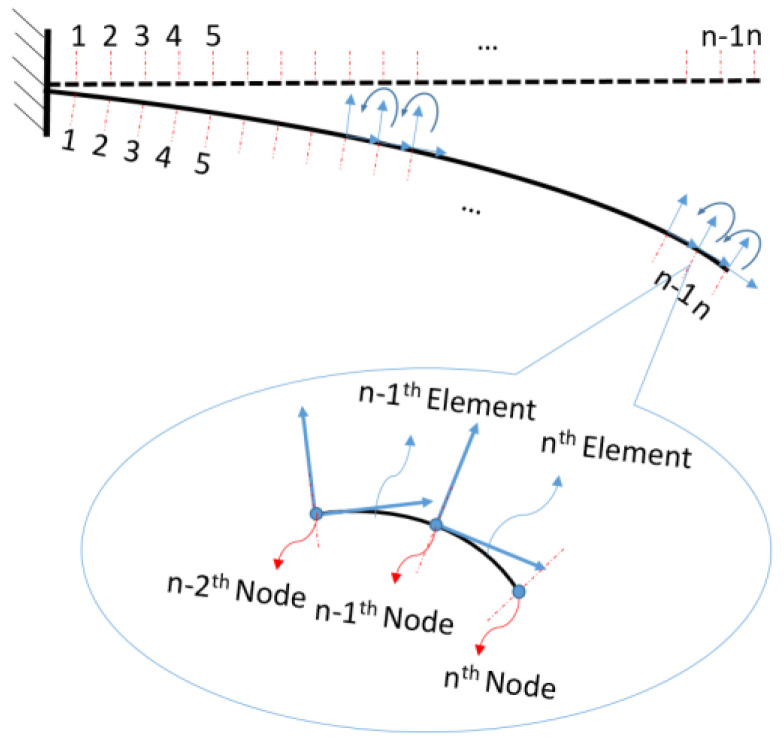
Element map for the coordinate transformation method.

The relation between the local strain and local curvature is given by Equation (4).

(4) $$\varepsilon_{n}=\frac{Td\theta_{n}}{2\Delta L}$$

where T, ΔL and $d\theta_{n}$ are the thickness of the beam, sensing length and change in rotation respectively for any sensing part n . The measured strain in each sensing part along the surface of the structure can then be translated into the local curvature of each part via Equation (4). As can be seen from Equation (4), the rotation of each element can be calculated directly from the measured strain on this sensing part, while in discrete sensing, each element’s rotation is calculated by adding two half rotations at its two adjacent measuring points. For the CTM in distributed sensing, the element map of the structure is shown in Figure 8. After translating the measured strain into rotations, ρ (i.e., curvature) and  θ from the local coordinate of the element will be translated into the previous element’s (or sensing part) local coordinate system and finally to the global coordinate via recursion Equations (5) and (6), where $\left(x_{n}^{m},y_{n}^{m}\right)$ represents the coordinate of the nth node in the mth element’s local coordinate system. The mth element’s local coordinate system is a system in which the origin of coordinate located at the mth element’s left end and its *x*-axis is the tangent line at the mth element’s left end. For example, $\left(x_{5}^{4},y_{5}^{4}\right)\;$, represents the location of the 5th node in the 4th element’s local coordinate system. $\;\left(x_{n}^{1},y_{n}^{1}\right)$ represents the nth node’s coordinate in the 1st element’s coordinate system which is the global coordinate system of the cantilever beam. By plotting all the nodes’ coordinate in the global coordinate system $\left(x_{0}^{1},y_{0}^{1}\right)\;\mathrm{to}\;\left(x_{n}^{1},y_{n}^{1}\right)$, the shape of the structure will then be configured. In some cases, when the 1st element’s coordinate system is not the global coordinate system an extra coordinate transformation is required to transform the $\left(x_{0}^{1},y_{0}^{1}\right)\;\mathrm{to}\;\left(x_{n}^{1},y_{n}^{1}\right)$ into $\left(x_{0}^{0},y_{0}^{0}\right)\;\mathrm{to}\;\left(x_{n}^{0},y_{n}^{0}\right)$ which is the coordinate in the global system.

The recursion formulas to calculate the shape in the CTM are the following:

(5) $$\left(x_{n}^{n},y_{n}^{n}\right)=\left(\frac{T}{2\varepsilon_{n}}\sin\left(\frac{2\Delta L\varepsilon_{n}}{T}\right),\frac{T}{2\varepsilon_{n}}\left(1-\cos\left(\frac{2\Delta L\varepsilon_{n}}{T}\right)\right)\right)$$

…

(6) $$\left(x_{n}^{m},y_{n}^{m}\right)=\left(\begin{array}{c} x_{n}^{m+1}\cdot \cos\left(\frac{2\Delta L\varepsilon_{m}}{T}\right)-y_{n}^{m+1}\cdot \sin\left(\frac{2\Delta L\varepsilon_{m}}{T}\right)+\frac{T}{2\varepsilon_{m}}\cdot \sin\left(\frac{2\Delta L\varepsilon_{m}}{T}\right),\\ x_{n}^{m+1}\cdot \sin\left(\frac{2\Delta L\varepsilon_{m}}{T}\right)-y_{n}^{m+1}\cdot \cos\left(\frac{2\Delta L\varepsilon_{m}}{T}\right)+\frac{T}{2\varepsilon_{m}}\cdot \left(1-\cos\left(\frac{2\Delta L\varepsilon_{m}}{T}\right)\right) \end{array}\right)$$

where, $\varepsilon_{n}$ represents the average strain on element n.

**Figure 9:**
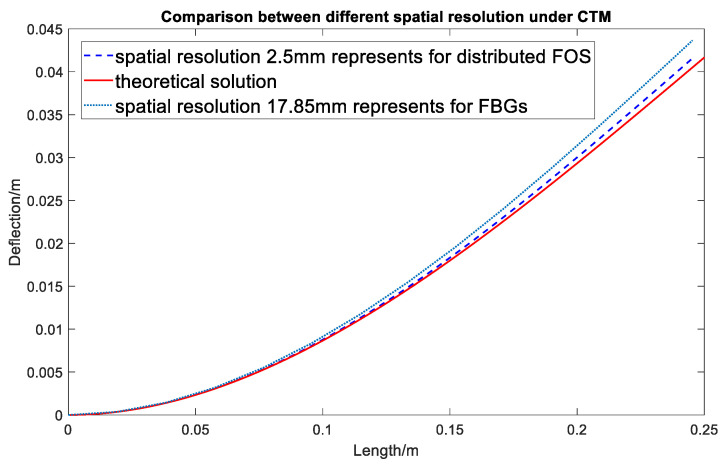
Comparison between different spatial resolutions in coordinate transformation method.

After the derivation of the CTM algorithm, a numerical simulation is carried out in which the CTM with different spatial resolutions is considered and compared with theoretical results to see how much influence the spatial resolution will have on the accuracy of the shape sensing. A cantilever beam of 250 mm length is considered, where the strain variation starting from its fixed end is 2000–0  με. The simulated strain matrix is input to the CTM algorithm with two different spatial resolutions: 2.5 mm and 17.85 mm to predict the deformed shape of the beam. These two spatial resolutions are chosen here because 2.5 mm is the spatial resolution of the RBS utilized in this research, while 17.85 mm is similar to the maximum spatial resolution achieved by FBG sensors in shape sensing in the literature [27]. Figure 9 shows the comparison between the CTM-configured shape and the theoretical calculated result for two different scenarios: simulated FOS and simulated FBG to compare the accuracy of the shape sensing using point sensing (FBG) and continuous lateral sensing (distributed FOS). The theoretical result is the analytical solution based on the Euler–Bernoulli beam assumption [28].

The results of shape sensing with the CTM with distributed FOS on one side of the beam was derived and validated in this section. In the next section, the SDEM for distributed FOS is derived.

#### 3.2.2. SDEM for Distributed FOS

**Figure 10:**
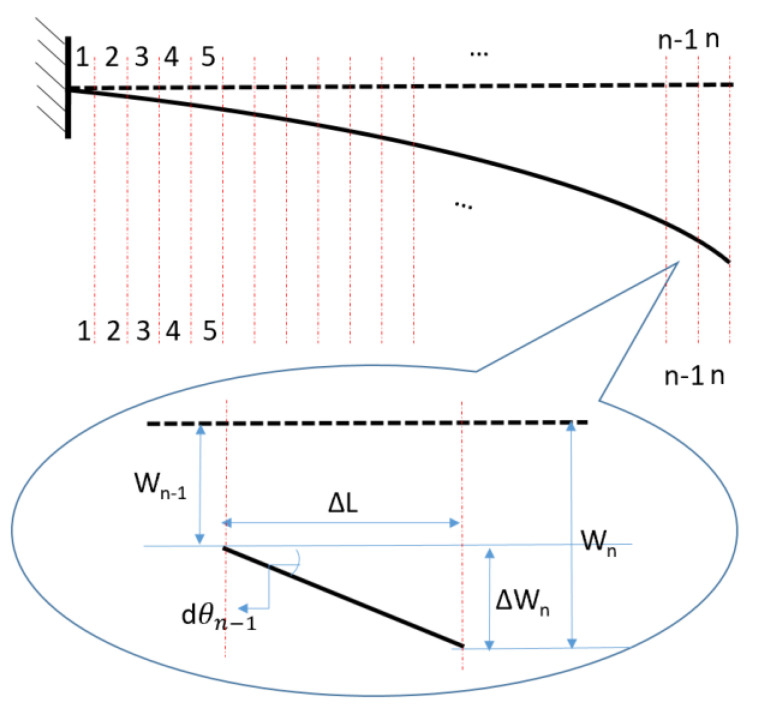
Element map for strain-deflection equation method.

The element map of the SDEM for distributed FOS is shown in Figure 10. Assuming that there is no displacement in axial direction, the rotation angle of each element is $d\theta =\frac{\Delta W}{\Delta L}$, where ΔW is the vertical deflection of each element.

By inputting the measured strain into Equations (7)–(12), the shape of the structure can then be configured. It starts from the first node’s rotation and deflection, which is $\theta_{1}=w_{1}=0$ for the cantilever beam.

(7) $$\theta_{2}=\frac{2\varepsilon_{x}\left(1\right)\Delta L}{T}+\theta_{1}$$

(8) $$W_{2}=\Delta L\times \left(\frac{2\varepsilon_{x}\left(1\right)\Delta L}{T}+\theta_{1}\right)+W_{1}$$

(9) $$\theta_{3}=\frac{2\varepsilon_{x}\left(2\right)\Delta L}{T}+\theta_{2}$$

(10) $$W_{3}=\Delta L\times \left(\frac{2\varepsilon_{x}\left(2\right)\Delta L}{T}+\theta_{2}\right)+W_{2}$$

(11) $$\theta_{n}=\frac{2\varepsilon_{x}\left(n-1\right)\Delta L}{T}+\theta_{n-1}$$

(12) $$W_{n}=\Delta L\times \left(\frac{2\varepsilon_{x}\left(n-1\right)\Delta L}{T}+\theta_{n-1}\right)+W_{n-1}$$

**Figure 11:**
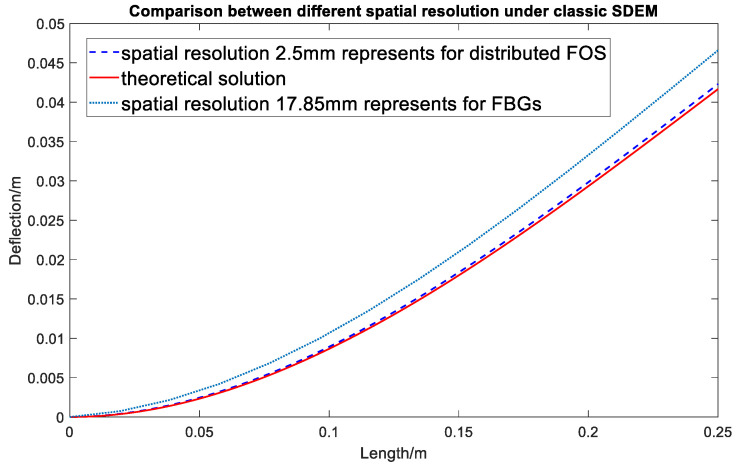
Comparison between different spatial resolutions in SDEM.

Similar to the CTM, the SDEM with different sensing spatial resolutions is applied in a simulated example presented previously to investigate the influence of the spatial resolution in the shape sensing accuracy. Figure 11 presents the results of the SDEM-predicted shape with RBS’s spatial resolutions (continuous lateral sensing) and FBG’s spatial resolutions (point sensing) against the theoretical result.

**Figure 12:**
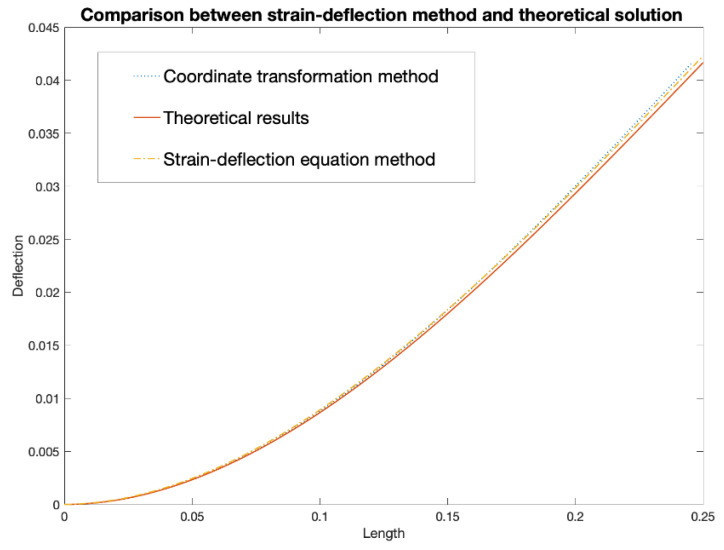
Comparison between CTM and SDEM algorithms.

The simulation results in Figure 9 and Figure 11 show that the increasement in the FOS’s spatial resolution can reduce the error by around 5%. However, the difference between these two methods is not noticeable for a simple point load (shown in Figure 12). Compared to the SDEM method, the CTM is originally used for large displacement, therefore, it takes the axial displacement caused by the deflection into consideration. This leads to the shortening of the beam in the axial direction which provides a more realistic deformed shape. However, for an example of a wind turbine blade where the maximum strain goes up to around 5000 με, the effect of the axial displacement is not obvious and can actually be ignored, especially when the RBS’ high spatial resolution has already reduced the error. Its improved accuracy does not overweight its increased complexity in the algorithm, and for structures under complex and large loading, the advantage of the CTM cannot be ignored. In the next section, the algorithm optimization is introduced in order to improve the system’s performance under complex loading, which is the more realistic and generalized scenario.

It is worth mentioning that both of the derived algorithms have calculated the intermediate variable angle of each element $\theta_{n}$. While in previous SDEM applications in discrete sensing, the rotation was not included. In load monitoring, once the external vertical loading is configured, the angle can be used to separate the vertical component in each element to calculate the total lift the structure has.

## 4. Distributed FOS Algorithm Optimization

**Figure 13:**
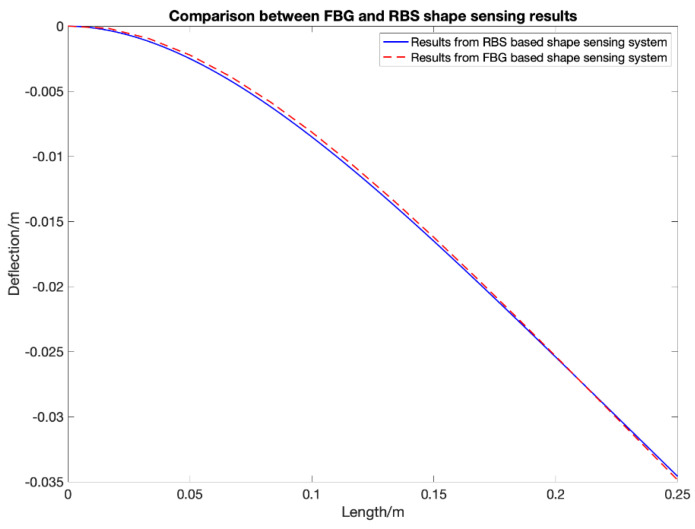
Comparison between fiber Bragg grating sensor and Rayleigh backscattering sensors under uniform distributed loading.

**Figure 14:**
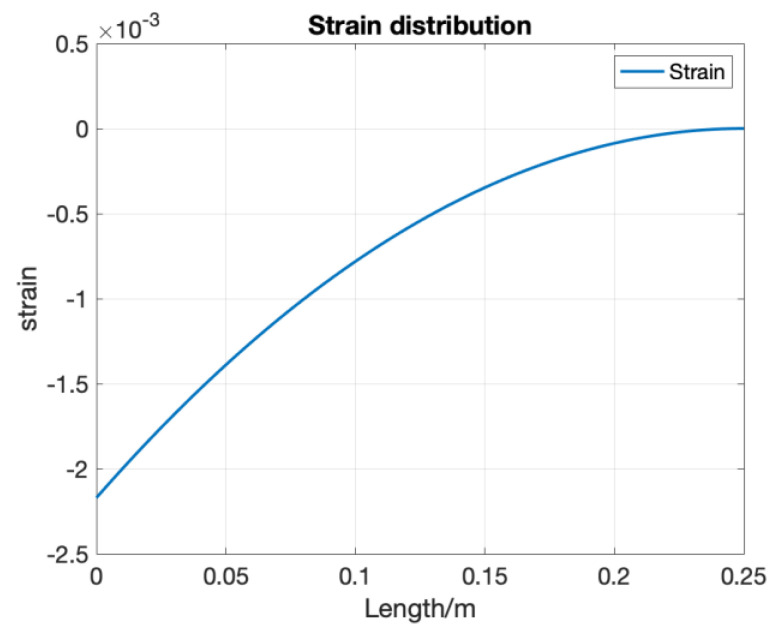
Strain distribution when structure is under uniform distributed loading.

The algorithm for shape sensing with distributed FOS was derived in the last section. However, using the uniform average strain as the strain distribution within each element part will cause an error, since in real situations, external loading is more likely to be distributed and non-uniform. Figure 13 shows the comparison between FBG-based and RBS-based shape sensing results when the structure is under the strain distribution shown in Figure 14 which represents the structure under a uniform distributed loading. Although the RBS system has a higher spatial resolution, its accuracy in shape sensing is still similar to the FBG’s system. This shows that using the uniform average strain as the strain distribution within each element will lead to an error which counteracts the improvements made due to a higher spatial resolution.

Furthermore, for FBG and BBS, a malfunction is more likely to be an error in the measured spectrum, since the distortion of the spectrum will lead to the difficulties in the centre wavelength configuration. Therefore, Nishio [15] proposed to apply the reliability calculation and curve fitting method after measuring the strain profile to decrease the error caused by the BBS system’s low spatial resolution and spectrum distortion. By contrast, in terms of RBS, as mentioned in Section 2, the sensing part is identified by cross-correlation analysis. A distorted spectrum is more likely to lead to the failure of identifying the correct location, which means “no signal” or “not a number” in the system output. A method to increase the system’s robustness is required to make sure the system still performs well when some sensing part cannot be identified.

In addition, for shape sensing of large structures such as aircraft wings or turbine blades, the self-weight of the structure will cause a noticeable strain in both axial and bending directions. In this scenario, the axial displacement should be taken into consideration. In the next section, modified algorithms are proposed which enable the RBS-based shape sensing system to have a higher accuracy under complex or combine loading (tension and bending) and a higher robustness in case of sensor failure.

### 4.1. Complex Loading and Sensor Malfunction

To increase the accuracy of the shape sensing as well as increasing the robustness of the sensing system, the interpolation method with spline interpolation of order three is proposed. The third order interpolation is more suitable for non-uniform external loading distribution. Furthermore, if one or more sensors within the optic fiber fails, the error caused by the malfunction is minimized as well.

**Figure 15:**
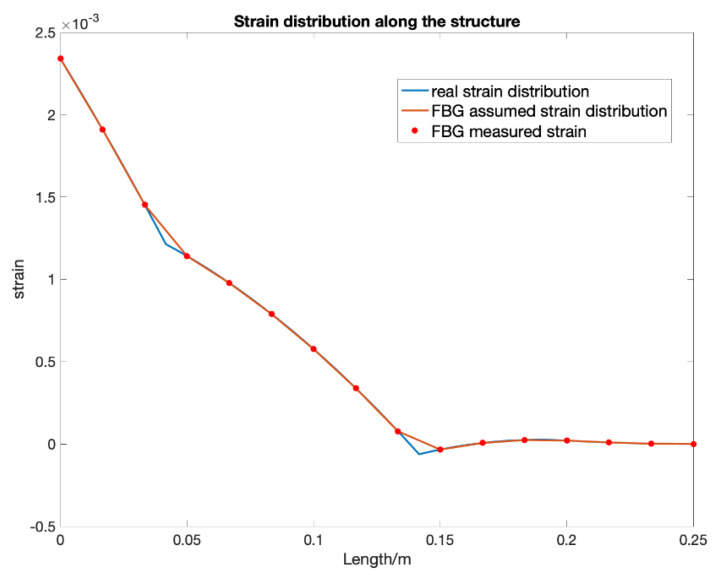
Strain distribution along the cantilever for complex loading.

**Figure 16:**
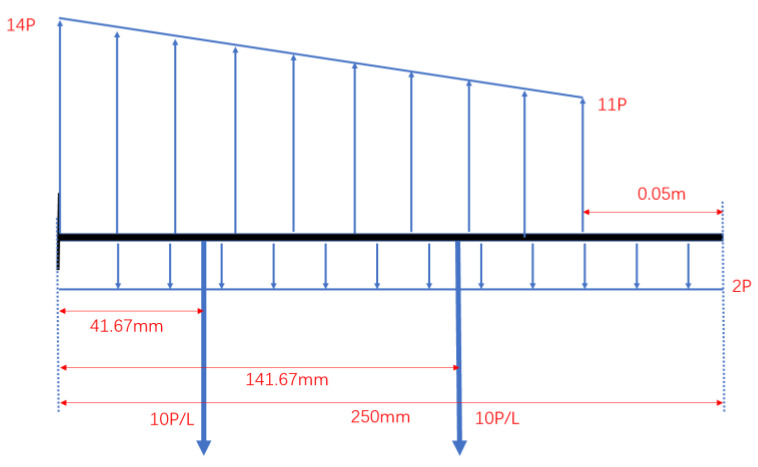
External loading representative of air-foil.

**Figure 17:**
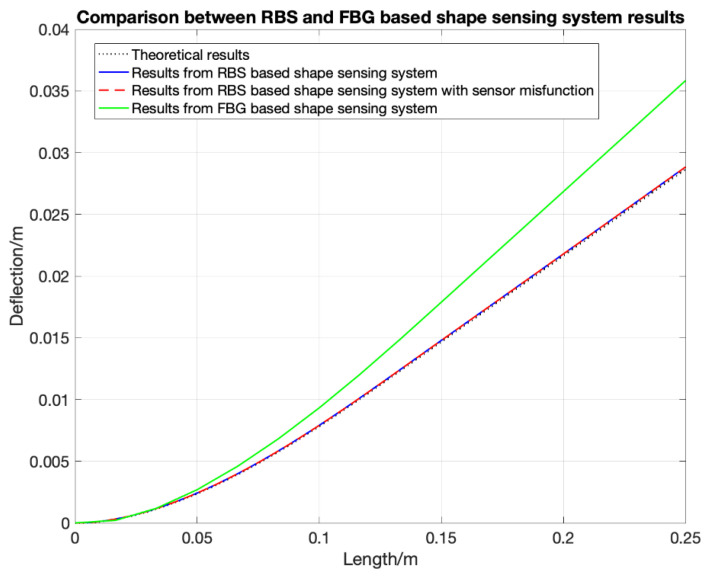
Shape sensing results from the RBS sensor and the FBG sensor.

Similar to the previous sections, a numerical simulation is carried out to validate the shape sensing system’s performance under a more realistic air-foil loading, where a combination of distributed loads and point loads are applied, see Figure 15. The real strain distribution is the result calculated from the Euler–Bernoulli beam theory. The cantilever specimen is of the same size as the one presented in Section 3.2., the resulting strain distribution along the beam is shown in Figure 16 and is no longer linear. The loading is a more realistic representation of a wing structure, where the distributed loading of different amplitudes are applied at the top and bottom of the cantilever, with 2 concentrated loads at 41.67 mm and 141.67 mm with magnitude 10 PL (P is a unit of pressure load), which represents the weight of two engines. RBS sensors are bonded on the top surface through the whole structure. The RBS sensing system has a spatial resolution of 2.5 mm, while FBG sensors are bonded at fifteen equidistance points. The predicted deformations are shown in Figure 17 with the solid blue and green lines representing the shape sensing based on continuous (RBS) and point sensing (FBG), respectively. After that, the shape reconstruction algorithm is applied again but this time considering that the three sensors have failed: 3rd to 5th in RBS’s sensing part. The results are then shown in Figure 17 with the dashed red lines. The theoretical solution is shown with a black dash line which is the analytical solution calculated by MATLAB from the ordinary differential equation of the beam. The simulated results show that when applying the SDEM algorithm with the FBG sensor in a real air-foil loading, the error can go up to around 30% compared to the theoretical solution. While the error with the RBS sensing system is within 1% even through only two data are interpolated between two adjacent measured data. Little error is introduced when three sensing parts failed to measure any data. These results validated that after the interpolation, the continuous lateral sensing (RBS) with a 3rd order interpolation function has a better performance than the current shape sensing systems with FBG under real air-foil loading. Furthermore, a higher robustness is achieved when using interpolation. The example studied in this section only considered transverse loading. To fully assess the benefits of the two different sensing systems, their performance under combined axial and transverse loading is assessed in the next section.

### 4.2. Combined Loading

When the structure is under combined loading, which involves both axial (tension or compression) and transversal (bending) loading, taking the axial displacement into consideration can improve the accuracy of the shape sensing. In that case, the rotation–deflection relationship $d\theta =\frac{\Delta W}{\Delta L}$ in Equations (7)–(12) has been changed into $d\theta =\frac{\Delta W}{\Delta L\left(1+\varepsilon_{a}\right)}\;$, where $\varepsilon_{a}$ is the axial strain within the optic fibre. It can be measured by bonding the FOSs on both sides of the structure. Therefore, the strain caused by bending can be calculated as: $\varepsilon_{x}=\frac{\varepsilon_{top}-\varepsilon_{bottom}}{2}\;$, while the strain caused by tension or compression can be calculated as: $\varepsilon_{a}=\frac{\varepsilon_{top}+\varepsilon_{bottom}}{2}$ in combination will result in Equation (13) for obtaining the transverse deflection of any sensor part n:

(13) $$W_{n}=\Delta L\left(1+\varepsilon_{a}\left(n-1\right)\right)\times \left(\frac{2\varepsilon_{x}\left(n-1\right)\Delta L}{T}+\theta_{n-1}\right)+W_{n-1}$$

**Figure 18:**
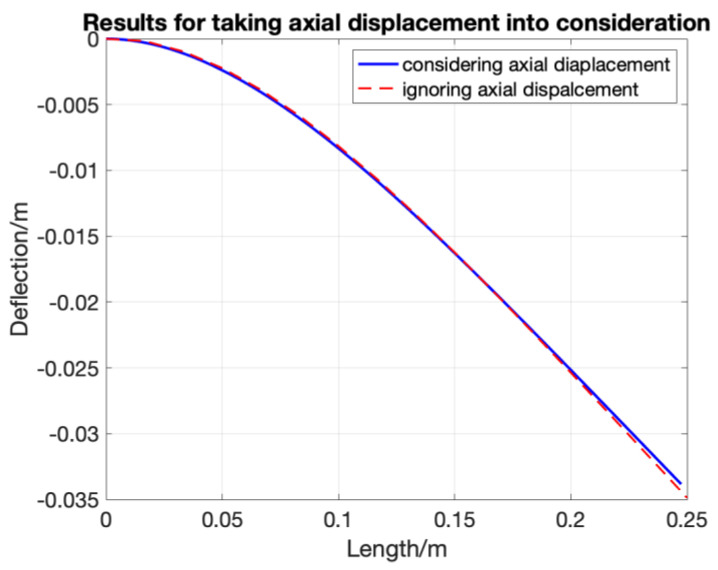
Comparison between different spatial resolutions in modified SDEM.

By modifying the algorithm, the axial strain is taken into consideration. The numerical simulation result for considering an axial strain with a magnitude of 2000 με and a uniform distributed loading, which is same as the one shown in Figure 14, is presented here, in Figure 18.

The results show that when considering the axial displacement, the error in the deflection direction can be reduced by maximum 4%. This is not a noticeable improvement.

In this section, an improved algorithm when under more realistic air-foil loading or combine loading is proposed. The simulated results show that the optimized distributed (RBS-based) shape sensing system has a higher performance under realistic loading air-foil loading compared to point sensing (FBG-based) system. In next section, an experiment is carried out and reported to validate and verify the RBS-based shape sensing system.

## 5. Experimental Validation

To validate the application of RBS in shape sensing, an experiment is carried out. The RBS was bonded onto the surface of a composite cantilever specimen to measure the strain distribution along the length of the beam. The shape of the specimen was then configured from the RBS-measured strain. The configured shape was compared to the deformed shape measured by an optical camera for validation and verification.

### 5.1. Specimen Preparation

**Figure 19:**
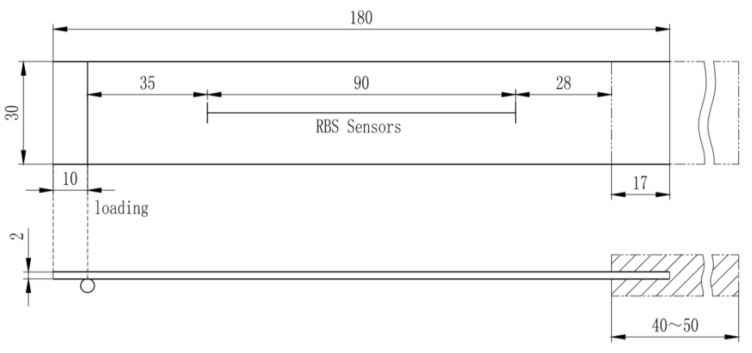
Specimen geometry and test setup.

The CFRP specimen was manufactured from thermoset prepreg (hexcel 914C-TS-5) as the host structure with the composite layup of: [0/45/−45/90/90/−45/45/0]_S_. The size of the specimen is 180 mm×30 mm×2 mm. RBS was placed in the middle of the lower surface of the specimen along the axial direction. There are 36 sensing parts within the optic fibre, each with a sensing length of 2.5 mm. The demodulation system used in this experiment is a commercial system Luna ODiSI B. One end of the specimen was fixed onto a cantilever beam fixture while a concentrated loading was applied at its other end. The location of the sensors as well as the initial loading location and boundary condition is shown in Figure 19. The bottom cantilever beam fixture was fixed on the base of the loading machine. The loading head was a cylindrical metal stick. It was not fixed onto the specimen. With the increasing of deflection, the loading line on the specimen will move to the right slightly. The RBS sensed the strain distribution in the middle part of the specimen. The strain distribution along the whole structure was then extrapolated from measured strain profile.

### 5.2. Experimental setup

**Figure 20:**
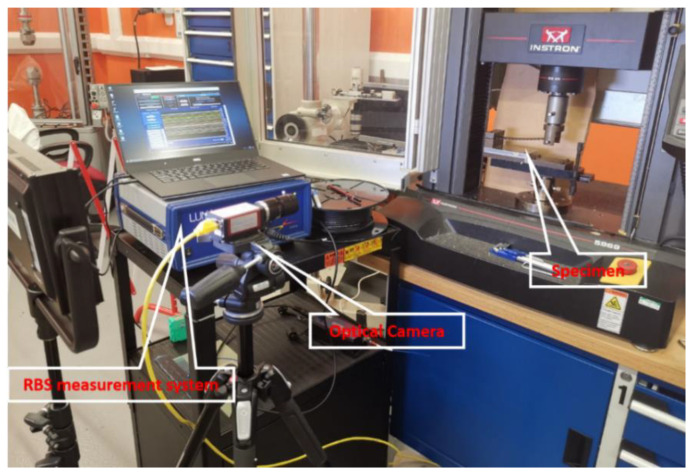
Experimental setup.

**Figure 21:**
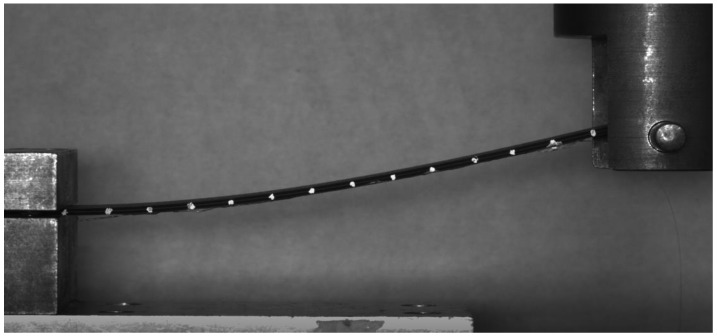
Experimental shape sensing with optical camera.

The setup of the experiment is shown in Figure 20. The loading machine added an external load onto the specimen, causing the specimen to bend. To measure the deformed shape of the beam, 13 points were marked on the side of the specimen with intervals of 10 mm (the rightmost point is used to identify the loading head and therefore considered as an observation point), as shown in Figure 21. The displacement of these points were measured by an optical camera and compared with the result calculated from the RBS’ measured strain distribution for validation.

## 6. Discussion

**Figure 22:**
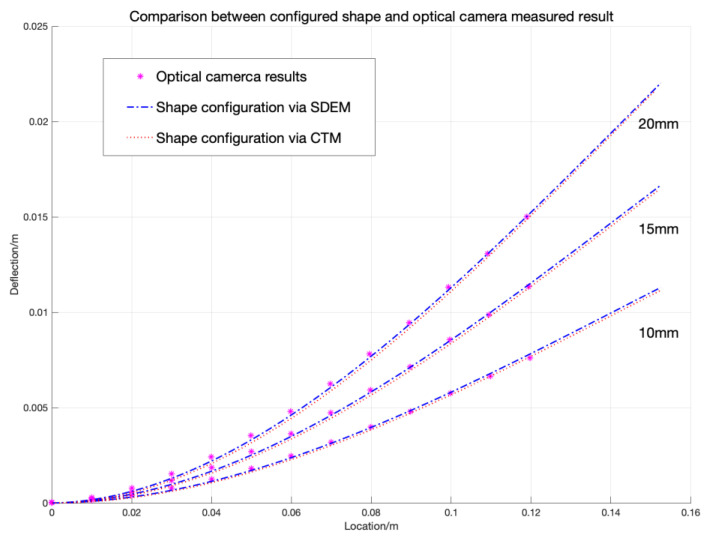
Experimental and simulated results.

The shape configuration resulting from the RBS strain distributions with both CTM and SDEM algorithms are shown in Figure 22 with red dotted line and blue dot-dash line, respectively. The predicted deformed shapes under three different conditions are compared, in which the displacements of the loading head are 10 mm, 15 mm, and 20 mm, respectively.

The comparison between distributed the FOS-based shape sensing and optical camera results shows that both the strain-shape algorithms predicted the deformed shapes with high accuracy, and they match well with the optical camera results, which validate the RBS-based shape sensing system. It is worth noting that the load profile in this example is a constant point load. However, in the previous sections it was demonstrated that the complexity of the loading (e.g., combined load, non-uniform distributions) will change the accuracy of the prediction on the basis of the resolution of the RBS sensors. The objective of this research was also to investigate this effect experimentally. However, the current experimental set up was not able to apply combined loading simultaneously. This will be considered in the next step of the research to improve the reliability and robustness of the algorithm.

## 7. Conclusions

By comparing the spatial resolution of different distributed FOS, the most suitable distributed FOS, RBS, was selected which has the advantage of both continuous lateral shape sensing and high spatial resolution. RBS is then applied in shape sensing to improve current FOS-based shape sensing system’s performance. The RBS’s strain sensing accuracy was validated by an experiment comparing it with strain gauges. After that, two shape sensing algorithms (CTM and SDEM) based on the distributed FOS’ input strain data were derived and tested. A comparison between the two algorithms were made, which showed that the error between the two algorithms for small deformation is negligible. The algorithm was then optimized according to the distributed FOS’s feature, to make it applicable to complex loading situations and to improve its accuracy in case of sensor failures. After that, numerical simulations were carried out to validate the algorithms’ accuracy. The simulations showed that compared to the FBG-based system, the RBS-based system has a better performance in configuring the shape when the structure is under complex loading or combine loading.

Finally, an experiment was carried out, in which the RBS sensors were used to configure the shape of a composite cantilever-beam-like specimen under concentrated loading. The configured shape was then compared with the deformed shape measured by an optical camera. The experimental results show that both shape sensing algorithms predict the shape comparably well with the optical camera measured result.

## Figures and Tables

**Table 1:** Comparison between different kinds of distributed fiber optic sensing systems.

Theory	Measurable (T/ε )	Sensing Range	Spatial Resolution	Strain Resolution	Temperature Resolution	Reference
Rayleigh (OTDR)	T/ε	1−2 km	0.5 m	n/a	n/a	[17]
Rayleigh (OFDR)	T/ε	70–100 m	5 mm	1	0.1	[18]
Brillouin (BOTDA)	T/ε	10 km	1.5 m	2	0.1	[19]
Brillouin (BOTDR)	T/ε	45 km	5 m	2	0.1	[19]
PPP-BOTDA	T/ε	2 km	2 cm	20	n/a	[20,21,22]
BOFDA	T/ε	9 m/11 km	3 cm/1.4 m	30	1.8	[23]
Raman	T	20 km	1–2 m	n/a	<1	[24]
